# Effects of competition and predation risk from a life history intraguild predator on individual specialisation

**DOI:** 10.1111/1365-2656.70090

**Published:** 2025-06-29

**Authors:** Marine R. A. Richarson, Travis Ingram

**Affiliations:** ^1^ Department of Zoology University of Otago Dunedin New Zealand; ^2^ Present address: Department of Conservation Dunedin New Zealand

**Keywords:** antagonistic interactions, diet, habitat use, intraguild predation, intraspecific variation, invasion, mesocosm, niche shift

## Abstract

1. Individuals can deploy a variety of ecological and behavioural strategies to obtain resources, often using only a subset of the total resource diversity used by their population. This phenomenon of individual specialisation (IS) is nearly ubiquitous across taxa and has the potential to affect population dynamics and ecosystem processes. Pairwise antagonistic interactions such as competition and predation can influence the degree of IS in a population, but little is known about the combined effects of multiple simultaneous interaction types between species, including intraguild predation (competition and predation from a single antagonist).
2. We address this gap by asking how the combination of competition and predation risk from an invasive intraguild predator—Eurasian perch *Perca fluviatilis—*impacts the degree of dietary and habitat IS in a native New Zealand fish, the common bully *Gobiomorphus cotidianus*. Bullies exhibit a generalised diet at the population level and compete for benthic and pelagic prey with juvenile perch, while also being subject to predation by larger perch.
3. We used a mesocosm experiment to explore how competition from young‐of‐year perch and perceived predation risk from sub‐adult perch influence IS within bully populations. Over a 3‐month period, we monitored individual habitat use and used serial gastric lavage to sample time‐integrated individual diets.
4. We found that the presence of juvenile perch was associated with a decrease in dietary IS associated with a shift to more benthic feeding, while habitat IS was affected by an antagonistic interaction between competition and predation whereby presence of small perch negated a negative effect of large perch on IS.
5. This study demonstrates the importance of considering multiple interaction types when evaluating how interspecific interactions influence individual variation within populations.

## Graphical abstract

Individual niche specialisation is widespread within animal populations and can have important ecological consequences. Predation and competition are known drivers of individual specialisation, but their combined effects are unstudied. This mesocosm experiment shows that competition and predation from a single antagonist can have variable effects on individual specialisation in diet and habitat of a freshwater fish.

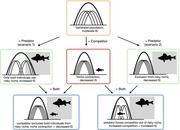

## 1 INTRODUCTION

Within populations, individuals deploy a variety of strategies to respond to spatial and temporal heterogeneity in their environment. Individual specialisation (IS) is defined as the use by individuals of only a subset of the total resources exploited by their population, resulting in intraspecific niche partitioning (Bolnick et al., 2002). The degree of IS within populations can be determined by a number of factors, including the diversity of available resources and the extent of intraspecific variation in phenotype or behaviour (Bolnick et al., 2003). Antagonistic ecological interactions, particularly competition and predation, are often invoked as potential ecological drivers of IS (Araújo et al., 2011).

Strategies used to reduce the impact of competition or the risk of predation can affect ecological traits both at the population and individual levels, including prey preference (Kondoh, 2003) and foraging tactics (Stein et al., 2015). For example, population niche shifts and niche contraction associated with decreased niche partitioning have been observed in heterospecific assemblages in which individuals compete for the same pool of resources (Costa‐Pereira et al., 2018). Populations experiencing high interspecific competition often occupy narrower niches, which often leads to less variation among individuals and might facilitate species coexistence (Tran et al., 2015). On the other hand, predator presence might have density‐ and trait‐mediated (e.g. behavioural) effects on resource use and on the degree of IS in a prey population (Preisser et al., 2005). For example, predation can decrease population size and thus reduce the generally diversifying effect of intraspecific competition (Araújo et al., 2011). Meanwhile, behavioural strategies for predator avoidance often involve a trade‐off between food and safety. Predator‐induced behavioural changes such as less frequent feeding (Beleznai et al., 2015), avoidance of risky foraging areas (Cronin et al., 2004), changes in movement patterns (Beleznai et al., 2015; Cote et al., 2013) and other escape responses (Kristensen & Closs, 2004) may alter habitat use and foraging patterns, and ultimately change the degree of IS. There is empirical evidence for predator presence both suppressing (Eklöv & Svanbäck, 2006) and increasing (Sharpe & Chapman, 2014) the degree of individual diet specialisation in prey. While both competition and predation risk can influence IS, we know little about the combined effects of multiple interaction types.

Disentangling the interactive effects of co‐occurring antagonistic interactions is a long‐running issue in ecology (Schoener, 1974). It can be challenging as their interactive effects can depend on trait plasticity (Werner & Peacor, 2003) and local ecological factors (Baines et al., 2014). When both competitors and predators are present, the degree of IS in the prey population might result from a balance between diversifying and constraining factors, such as access to resources, intensity of competition from both conspecifics and heterospecifics (Cronin et al., 2004), and the costs of predator‐induced shifts in resource use (Hooker et al., 2017). Individuals of many species undergo dietary shifts through ontogeny (Arim & Marquet, 2004; Polis et al., 1989) and may therefore change from being a competitor of a second species as a juvenile to a potential predator as an adult. This life history intraguild predation (IGP) means that a single intraguild predator population may have variable impacts on the degree of IS in an intraguild prey population, depending on the relative abundance of different life stages of the intraguild predator. For risk‐averse individuals, the opportunity to occupy more sheltered habitat might depend on their competitive ability. On the other hand, divergent behavioural responses from competing prey species might counterbalance the effects of competition. If a prey species occupies a constrained niche as an outcome of interference competition, for example, differences in anti‐predator tactics from each competitor might increase spatial or temporal segregation between species, reducing competition intensity and potentially leading to niche expansion. Finally, predation might enhance the effects of competition by reducing the range of habitats a population occupies and thus further reducing its niche breadth. Overall, the outcomes of interacting predation and competition from a life history intraguild predator are likely to depend on how both competitors respond to the presence of the predatory size class.

**FIGURE 1:**
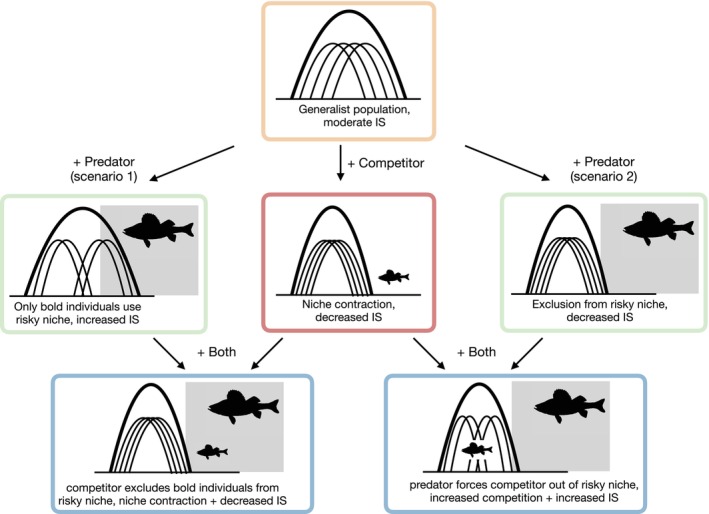
Hypothesised main and interactive effects of competition and predation on individual specialisation. Thick and thin curves represent population and individual niches in the focal species, with narrower individual niches relative to population niches indicating greater individual specialisation. Small fish silhouettes indicate interspecific competitor presence, while large fish silhouettes indicate predator presence along with a shaded area that represents niche spaces (habitats or diet items) that are riskier to use. Interspecific competition is typically expected to lead to niche contraction, while predation may lead to either greater specialisation by revealing behavioural differences among individuals (Scenario 1) or niche contraction if all individuals are excluded from risky habitats or diets (Scenario 2). These scenarios lead to different predictions about the combined effects of competition and predation (bottom row).

Here, we ask how life history IGP from introduced Eurasian perch *Perca fluviatilis* impacts the resource use diversity and the degree of IS in diet and habitat of a New Zealand native fish, the common bully *Gobiomorphus cotidianus*. We used a pond mesocosm experiment to factorially manipulate the presence of small perch as competitors and exposure to large perch as potential predators. We hypothesised that interspecific competition would lead to population niche contraction and reduced IS. We hypothesised that predation risk would either lead to increased IS if it led to greater habitat segregation among individuals, or to reduced IS if it led to niche contraction. We also hypothesised that competitor presence and predation risk would interact, with the form of the interaction dependent on how predation risk mediates behaviour and resource use of both competitors (Figure 1).

## 2 MATERIALS AND METHODS

### 2.1 Study species

The common bully is a habitat and dietary generalist that displays moderate levels of individual dietary specialisation in natural populations (Richarson, 2020). Bullies feed on a variety of benthic and pelagic invertebrate prey similar to the pool of resources used by juvenile perch. Bullies are also a common prey of piscivorous adult perch (Duncan, 1967; Griffiths, 1976) and exhibit anti‐predator strategies in response to perch chemical cues (Kristensen & Closs, 2004). The invasive Eurasian perch was introduced in New Zealand in 1868, and has spread to a wide range of lowland freshwater ecosystems across the country (McDowall, 1990). Perch undergo well‐documented ontogenetic diet and habitat shifts (Persson, 1988). Juveniles up to 80–120 mm total length (TL) inhabit pelagic habitats and initially feed mainly on zooplankton, while subadults increasingly feed on benthic macroinvertebrates and adults switch to piscivory at around 150–240 mm TL (Hjelm et al., 2000). Perch have documented impacts on the abundance, population dynamics and ecology of the common bully, both as a competitor and a predator (Goldsmith, 2004; Ludgate & Closs, 2003).

Both fish species were captured with seine nets on the shores of Lake Waihola (46°01′29.5″ S, 170°05′26.9″ E), a shallow coastal lake south of Dunedin, New Zealand, that contains large populations of common bully and perch, in November–December 2017. Common bullies (37–54 mm TL, *n* = 144 for experiment +24 spare), young‐of‐year perch (TL 52–68 mm, *n* = 18 for experiment +4 spare) and large perch (TL 170–250 mm, *n* = 6) were transported in well‐oxygenated lake water to the University of Otago's mesocosm facility in Sawyers Bay, Dunedin, and housed in 1200 L holding tanks separated by species and size class. Large perch were fed commercial fish pellets ad libitum while housed in their holding tanks. Prior to their introduction to the mesocosm tanks, each bully was lightly anaesthetised (0.025 mL/L solution of iso‐eugenol; AQUI‐S New Zealand Ltd., Lower Hutt, NZ), measured for TL and weighed to 0.1 g, before being marked with a subcutaneous Visible Implant Elastomer (Northwest Marine Technology, Inc., Shaw Island WA, USA) tag near the first dorsal fin to allow individual identification. All procedures used on fishes in this study were approved by the University of Otago Animal Ethics Committee (Animal Use Protocol 73‐17).

### 2.2 Mesocosm setup and experimental treatments

A total of 36 outdoor freshwater mesocosms were established in 1200 L plastic cattle troughs (0.65 m depth; 1.7 m upper diameter; Wilson Plastics, Palmerston North, NZ). They were filled with tap water, sprayed through a pressurised hose and allowed to sit for several days to allow chlorine to volatilise. A 1–2 cm deep layer of washed sand (<2 mm diameter) was then added to each tank, along with two ~180 cm^2^ patches of gravel (2–10 mm), several haphazardly distributed pebbles (50–150 mm) and one ~400 cm^2^ patch of macrophytes (*Myriophyllum triphyllum* and *Ruppia polycarpa*) to provide habitat heterogeneity. Overall, sand covered between 85% and 90% of the bottom of the tanks, while gravel patches represented 4%–9%, vegetation ~5% and pebbles ~2% of the available substrate. In each tank we placed a cage sized to temporarily house one large perch, consisting of a ~40‐cm diameter cylinder of plastic trellis mesh (mesh size 1.7 cm) placed upright from the bottom of the tank to above water level. The mesh size allowed passage by bullies and small perch, but not large perch. Finally, the tanks were seeded with zooplankton and benthic invertebrates and propagules. Zooplankton and mud were collected from multiple shallow ponds in the Waihola‐Waipori wetland complex using plankton nets and kicknets, then homogenised and distributed evenly between tanks. Invertebrate communities were then allowed to develop for 3 weeks until fish introduction.

Four bullies were introduced to each of the 36 mesocosms and left to acclimatise. Previous studies using these mesocosms have indicated that bullies at densities between 3 and 6 fish are affected by the competitive environment but still able to grow (Kerr & Ingram, 2021; Richarson, 2020). Nine mesocosms were randomly assigned to each of four treatments: control (bullies only); predation (bullies with periodic exposure to large perch); competition (bully with small perch); predation + competition (bullies with small perch and periodic exposure to large perch). The 2 × 2 design therefore aims to cross interspecific competition with perceived predation risk. One small perch was added to half of the tanks 2 days after bullies were introduced. Predation risk treatment and habitat observations started after 2 weeks of further acclimation. The predation risk treatment consisted of exposing the small fishes in the tanks to one large perch by gently releasing it into the cage and leaving it for 30 min before removal with a hand net. Consumption by large perch was not expected as bullies and small perch were able to swim out of the cage and likely had previously been exposed to large perch as potential predators. In the predator‐free treatments, the water in the cage was stirred with a clean hand net to mimic the disturbance associated with perch addition. The treatment was applied three times weekly for 16 weeks (22 December 2017 to 14 April 2018).

### 2.3 Diet and habitat monitoring

Once every 2 weeks during the experiment, bullies and small perch were recaptured with Gee minnow traps (set during the day and checked at least once per hour) and hand nets. Gastric lavage was used to repeatedly and non‐lethally sample stomach contents. Fish were housed for <30 min in a bucket filled with cool water from their tank. With the fish held securely with its head slightly angled downwards, a syringe was used to inject 1–3 mL of filtered mesocosm water at the back of its stomach via a thin polyethylene tube gently inserted through the mouth. The stomach contents and the water were regurgitated into an Eppendorf tube and immediately placed on ice until storage at −19°C. The fish was then returned to cool source water and returned to its tank after all fish had been recaptured, or after no further recaptures appeared likely (usually to a maximum of 3 h). Between one and five stomach content samples per fish were obtained over the course of the experiment and used for prey identification and analysis.

Stomach items were identified to the lowest taxonomic level possible. Common prey categories included chironomid larvae, daphniid and chydorid cladocerans, cyclopoid copepods, ostracods and gastropods. Body parts allowing identification of a taxon (e.g. heads of chironomid larvae) were included in the content counts, while unidentifiable objects or detritus were excluded. Prey items were aggregated at taxonomic levels (usually family or subfamily) in order to maximise the number of diet items that could be included, ensuring that partially digested prey could be identified and pooling rare taxa into higher taxonomic groups (Table 1).

**TABLE 1:** Summary of prey items found in stomachs of bullies and small perch (average percentages across all individuals across treatments).

Prey category	Taxon	Average percentage (%)	
		Bully	Small perch
Arachnida	Acari	0.3	0.6
Cladocera	*Ceriodaphnia* spp.	26.8	3.2
	*Chydorus* spp.	33.6	68.2
Copepoda	Cyclopoida	4.4	17
Diptera	Chironomidae larvae	24.1	9
	Chironomidae pupae	0.1	0.2
	Ceratopogonidae larvae	0.1	0
	Tanypodinae	0.4	0.1
Gastropoda	*Potamopyrgus* sp.	1	0
	Physidae	0.1	0
	Planorbidae	0.4	0
Ostracoda	Ostracoda	8.5	0.8
Other insects	Hemiptera (*Sigara* sp.)	0	0.1
	Odonata	0.2	0.7

Habitat use was recorded three times a week using a variant of instant focal surveys (Fulton et al., 2001). Tanks were approached cautiously by the observer to minimise any disturbance on fish in the focal tank or in adjacent tanks. Observations were performed for up to 5 min from the moment the first fish was spotted. Fish were identified based on their elastomer tag, and their habitat on first sighting was assigned to one of several microhabitat categories. These included water column (not on any substrate or structure), several substrate types (sand, gravel, pebble and vegetation) as well as three artificial habitat categories (tank wall, cage wall and cage floor).

Fish were checked three times weekly for mortality during habitat observations. During hot weather conditions in January 2018, fish mortality events led to variation in the identity and number (3 or 4) of bullies in tanks over time. Dead fish were replaced to maintain density until the 24 available replacement fish were used, but some tanks had enough mortality or low enough recapture rates that they could not be included in the analyses.

### 2.4 Data analysis

Tanks were removed from analyses if they did not contain at least three fish with at least three niche measures (diet items or habitat observations), either because of fish mortality or because fish were not observed or recaptured enough times. Because different tanks met this criterion for diet and habitat, the set of tanks analysed differed for the two data sets. The final diet data set included 21 tanks (5 control, 7 predation, 3 competition and 6 predation + competition), with a total of 84 fish with an average of 4.3 diet items per fish. The final habitat data set included 26 tanks (6 control, 7 predation, 6 competition and 7 predation + competition), with a total of 111 fish with an average of 13.5 observations per fish.

Separate analyses were carried out for the two niche measures: frequency of use of different diet items, and frequency of use of different habitat types. All statistical analyses were conducted using R 4.2.2 (R Core Team, 2022) with R Studio 2022.12.0. To test for resource use shifts between treatments, prey proportions for individual bullies were aggregated across sampling sessions, then averaged per tank, while habitat use proportions across all observations were also averaged per tank. Diet or habitat proportions were then converted to Bray–Curtis dissimilarities between tanks. Non‐metric multidimensional scaling (NMDS) was used to visualise the positions of the four treatments in multivariate resource use space. Juvenile perch were not included in the analyses, but separate NMDS were carried out including the two groups of juvenile perch (large perch present and absent) for context in interpreting bully results. The effects of predation risk, interspecific competition and their interaction on bully diet composition or habitat composition were tested using permutational multivariate analysis of variance (PERMANOVA) with the ‘adonis2’ function in the ‘vegan’ package, version 2.6‐4 (Oksanen et al., 2022). Homogeneity of dispersion was tested with the ‘betadisper’ function, and the contribution of specific taxa to the differences between groups was assessed using the ‘simper’ function with significance assessed using 999 permutations.

We calculated niche components to measure IS for both diet and habitat (Catry et al., 2014; Fodrie et al., 2015). The total niche width of a population (TNW) can be partitioned into two additive components: the average breadth of resources used by each individual (within‐individual component WIC) and the variation among individuals in average resource use (between‐individual component, BIC) (Bolnick et al., 2002; Roughgarden, 1972).

$$\mathrm{WIC}=-\sum_{i}p_{i\cdot }\left(-\sum_{j}p_{\mathrm{ij}}\;\ln\;\left(p_{\mathrm{ij}}\right)\right),$$

$$\;\mathrm{BIC}=\sum_{i}p_{i\cdot }\;\ln\left(p_{i\cdot }\right)-\sum_{j}q_{j}\left[-\sum_{i}\gamma_{\mathrm{ij}}\;\ln\left(\gamma_{\mathrm{ij}}\right)\right],$$

$$\mathrm{TNW}=-\sum_{j}q_{j}\;\ln\left(q_{j}\right).$$

Here, *p*_*ij*_ is the proportion of the resource use of individual *i* made up of resource type *j*; *q*_*j*_ is the proportion of the population resource use made up of resource type *j*; *p*_*i*·_ is the proportion of the population's total resources used by individual *i*; and 𝛾_*ij*_ is the proportion of the population's use of resource *j* accounted for by individual *i*. The index of IS WIC/TNW estimates how much of the population niche is made up of variation within individuals. We use the more intuitive complement 1‐WIC/TNW, so that values approaching 1 indicate that most variation is among individuals (high IS), while values approaching 0 indicate that most variation is within individuals (low IS) (Roughgarden, 1972).

We calculated WIC, BIC, TNW and 1‐WIC/TNW with the ‘RInSp’ package, version 1.2.5 (Zaccarelli et al., 2013), using individual diet proportions (aggregated across samples) or habitat proportions, and using summed individual data to calculate population resource proportions. A Monte Carlo resampling procedure was used to evaluate whether the observed 1‐WIC/TNW in each mesocosm population was higher than expected by chance, indicating statistically significant IS. This procedure generates a null distribution of 1‐WIC/TNW expected if individuals stochastically sample resources in proportion to their use by the population.

We used linear models to test the effects of predation risk, interspecific competition and their interaction on the IS index WIC/TNW, as well as the niche components WIC, BIC and TNW, for both diet and habitat. As the design was unbalanced due to the removal of failed replicates, we first used an ANOVA test using Type III SS with the ‘Anova’ function in the ‘car’ package (Fox & Weisberg, 2019) to test for an interaction between interspecific competition and predation. In cases where there was no significant interaction, we used a Type II SS to test for main effects of interspecific competition and predation. Where any significant effects were present, we used the ‘emmeans’ package to carry out post‐hoc pairwise comparisons between the four treatments. Effect sizes were estimated by calculating partial eta squared with the ‘heplots’ package (Friendly et al., 2023). Model residuals were visually checked for normality and homoscedasticity.

## 3 RESULTS

### 3.1 Population diet

Number of prey items sampled per individual bully ranged from 0 to 334, with an average ± standard deviation of 10.8 ± 33.3 prey items. For perch, an average of 71.8 ± 120.9 prey items were sampled per individual (range 0–716). In a single sampling session, individual fishes were found to prey on a maximum of 8 taxonomic groups, with an average of 1.2 ± 1.5 in bullies and 3.0 ± 2.3 in perch. Over all sessions, these values rose to 3.03 ± 2.1 and 5.9 ± 2.1 for bullies and perch, respectively. Seventeen of 109 bullies (15%) were removed from the dietary analysis as they were only ever sampled with empty stomachs.

**FIGURE 2:**
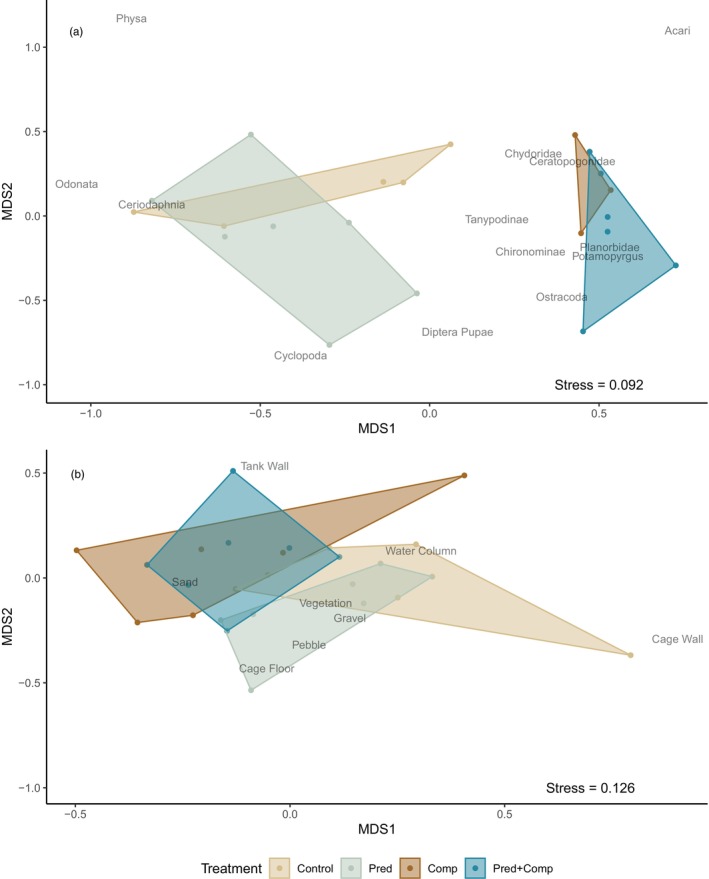
Difference in (a) diet and (b) habitat use of bully populations among treatments, illustrated using non‐metric multidimensional scaling (MDS) based on Bray–Curtis dissimilarity. Individual tanks are represented by points and grouped by convex hulls. Names indicate the centroid position of the ordination scores of each prey taxon or habitat type. Control: Bully only. Pred: bully + large perch. Comp: bully + small perch. Pred + Comp: bully + small perch + large perch.

Bullies and small perch consumed similar prey types. Diets included 14 taxonomic groups, with the most common prey types being cladocerans (chydorids from the Chydorinae and Aloninae subfamilies, and the daphniid *Ceriodaphnia dubia*), chironomid larvae, cyclopoid copepods and ostracods (Table 1). Non‐metric multidimensional scaling of the population mean diet compositions provided a good representation of the data (stress = 0.092). Intraspecific competition induced a significant shift in bully diet (*p* = 0.001, *R* ^2^ = 0.46; Figure 2a). This was driven by mesocosms with small perch having lower proportions of *Ceriodaphnia dubia* (0.43 vs. 0.01; *p* = 0.001) and higher proportions of chydorids (0.42 vs. 0.24; *p* = 0.040), chironomid larvae (0.38 vs. 0.20; *p* = 0.005) and ostracods (0.14 vs. 0.04; *p* = 0.011). There were no significant dietary shifts associated with predation risk or with the interaction between competition and predation risk (Table 2), and the assumption of homogeneity of dispersion among groups was met (*p* = 0.82).

**TABLE 2:** Effects of competition (Comp, small perch present), predation risk (Pred, large perch exposure) and their interaction (Comp × Pred) on bully diet and habitat use. Effects on composition were tested with a two‐way PERMANOVA with permutation‐based significance tests. Effects on diet components were tested with ANOVA, initially Type III sums of squares to test for an interaction term, and Type II sums of squares were used if the interaction was non‐significant.

Variable	Comp		Pred		Comp × Pred	
	*F*	*p*	*F*	*p*	*F*	*p*
Diet						
Composition	**15.5**	**0.001**	1.00	0.38	0.08	0.96
1‐WIC/TNW	**20.6**	**<0.001**	1.52	0.23	0.76	0.40
TNW	2.62	0.12	0.00	0.99	0.06	0.81
WIC	**14.5**	**0.001**	0.46	0.51	0.61	0.44
BIC^a^	**7.27**	**0.015**	0.11	0.74	0.06	0.81
Habitat						
Composition	**3.92**	**0.005**	0.97	0.43	0.65	0.67
1‐WIC/TNW	0.54	0.47	**7.79**	**0.011**	**4.93**	**0.037**
TNW^b^	4.03	0.057	0.00	0.98	3.17	0.089
WIC	**5.33**	**0.031**	0.66	0.43	0.16	0.70
BIC	2.83	0.11	**8.76**	**0.007**	**7.10**	**0.014**

*Note*: Significant effects are indicated in bold.

Abbreviations: BIC, between‐individual component; TNW, total niche width; WIC, within‐individual component.

^a^ BIC (Diet) was log‐transformed for analysis to improve normality of residuals.

^b^ TNW (Habitat) had left‐skewed residuals that could not be fixed by standard transformations.

### 3.2 IS in diet

**FIGURE 3:**
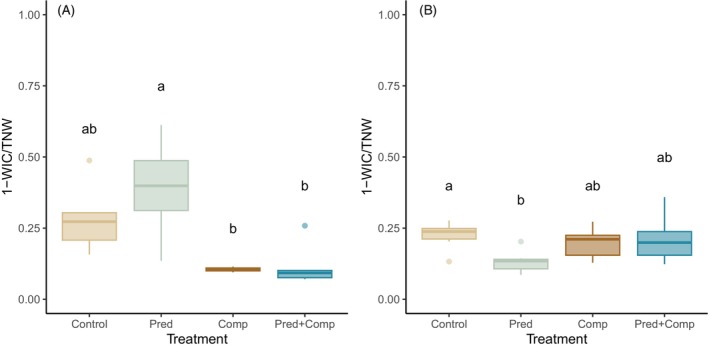
Index of individual specialisation 1‐WIC/TNW in (A) bully diets and (B) bully habitat use across treatments (Control = bullies alone; Pred = bullies and large perch; Comp = bullies and small perch; Pred + Comp = bullies and large perch and small perch). Higher 1‐WIC/TNW values indicate higher degrees of individual specialisation. Boxplots depict the median, first and third quartiles with whiskers extending up to 1.5 times the interquartile range. Treatments with different superscript letters differed significantly in a Tukey HSD post‐hoc test. TNW, total niche width; WIC, within‐individual component.

Over all treatments, values of 1‐WIC/TNW ranged from 0.07 to 0.61 (mean 1‐WIC/TNW = 0.25 ± 0.16 SD) which denotes low to moderate levels of IS in the mesocosm populations. Despite small population sizes, 17 of 21 mesocosm populations showed significant IS, while the null model of a population of stochastically foraging generalists could not be rejected in the other four. The degree of IS 1‐WIC/TNW was significantly affected by competition, with a strong effect size (*p* < 0.001, $\eta_{p}^{2}$ = 0.55; Figure 3A), while there was no main effect of predation risk or interaction between competition and predation risk (Table 2).

**FIGURE 4:**
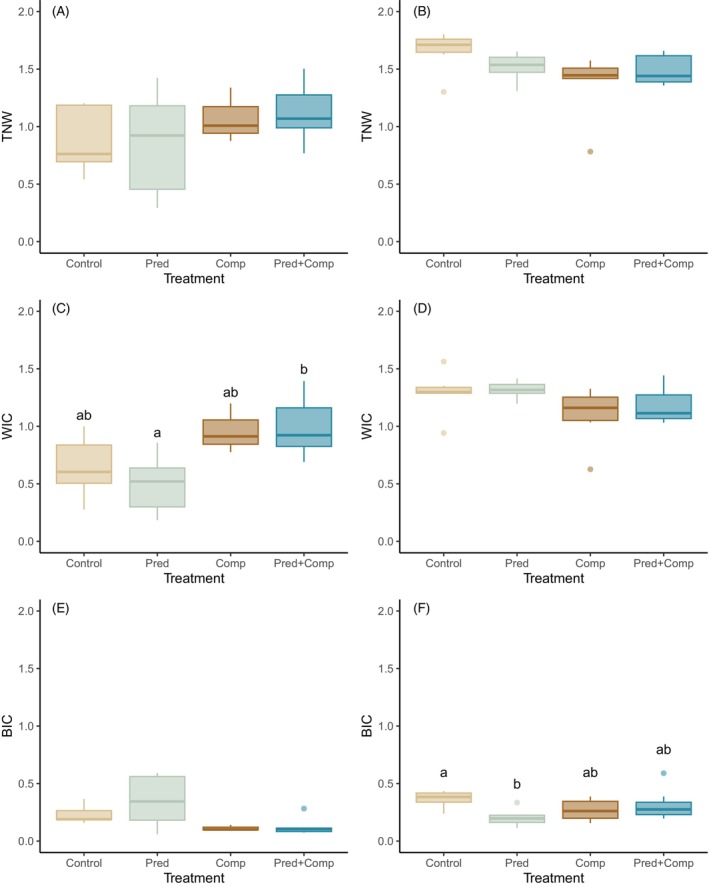
Niche components for diet (left column) and habitat (right column) across treatments (Control = bullies alone; Pred = bullies and large perch; Comp = bullies and small perch; Pred + Comp = bullies and large perch and small perch). Variables are population total niche width (TNW, A, B), average within‐individual variation (WIC, C, D) and between‐individual variation (BIC, E, F). Boxplots depict the median, first and third quartiles with whiskers extending up to 1.5 times the interquartile range. Treatments with different superscript letters differed significantly in a Tukey HSD post‐hoc test.

The TNW of mesocosm populations was not affected by either predictor (Figure 4A), but there were effects on the within‐ and between‐individual components of the dietary niche. Competition had a significant effect on WIC (*p* = 0.0014, $\eta_{p}^{2}$ = 0.46; Figure 4C), with higher individual niche breadths where small perch were present, while there were no significant effects of predation or the interaction. Competition also had a negative effect on BIC (*p* = 0.015) with a moderate effect size ($\eta_{p}^{2}$ = 0.30), and no significant effects of predation or the interaction (Figure 4E).

### 3.3 Population habitat use

The microhabitat types most commonly recorded for bullies were sand (43% of total observations), water column (21%) and tank wall (10%), with remaining microhabitats ranging from 3% to 7% of observations. In treatments in which they were present, small perch used the water column (27%) most, followed by the cage floor (25%), sand (20%) and vegetation (18%). Non‐metric multidimensional scaling of the habitat compositions provided an effective representation of the data (stress = 0.126).

Interspecific competition induced a significant shift in bully habitat use at the population level (*p* = 0.005, *R* ^2^ = 0.14; Figure 2b). Bullies in mesocosms with small perch present made less use of the cage wall (0.01 vs. 0.05 by proportion; *p* = 0.029) and more use of the outer tank wall (0.14 vs. 0.05; *p* = 0.008), as well as marginally less use of the water column (0.17 vs. 0.25; *p* = 0.055) and pebbles (0.06 vs. 0.10, *p* = 0.054). There was no significant effect of predation nor interactive effect with competition on population habitat use (Table 2), and the assumption of homogeneity of dispersion among groups was met (*p* = 0.70).

### 3.4 IS in habitat use

The degree of habitat specialisation among individuals was generally low, with an average 1‐WIC/TNW of 0.17 ± 0.03 over all treatments (Figure 3). In most tanks there was insufficient evidence to reject the null hypothesis of random habitat use, with only six tanks (two control, one competition and three predation + competition) having statistical evidence for significant IS in habitat based on randomisation tests. Nonetheless, there was variation among replicates in the estimate of 1‐WIC/TNW, and there were some statistical differences among treatments. Specifically, there was a weak but significant interactive effect of competition and predation on 1‐WIC/TNW (*p* = 0.037, $\eta_{p}^{2}$ = 0.182; Figure 3B). The interaction reflects a decrease in IS from control to predation treatments, but no decrease in either competition or predation + competition treatments.

Population habitat niche width (TNW) was not significantly affected by any predictor (Figure 4B), although there was a near‐significant negative effect of competition with small perch on TNW (*p* = 0.057, $\eta_{p}^{2}$ = 0.155). Competition did have a significant negative effect on the average individual habitat niche width WIC (*p* = 0.031, $\eta_{p}^{2}$ = 0.194; Figure 4D). In contrast, BIC was affected by a significant interaction between competition and predation (*p* = 0.014, $\eta_{p}^{2}$ = 0.244; Figure 4F). Patterns were similar to those for 1‐WIC/TNW, with BIC highest in control mesocosms, lowest in the predation treatment and intermediate in competition and predation + competition treatments.

## 4 DISCUSSION

With this mesocosm experiment, we demonstrated how the combination of competition and predation risk affect the dietary and habitat niche of bullies at the population and individual levels. The presence of small perch as interspecific competitors led to shifts in bully diet as well as reduction in the degree of IS. In contrast, while small perch also led to habitat shifts in bullies, IS in habitat instead showed an interactive effect of competition and predation risk. Our results suggest that predation risk and competition affect different components of a population's niche and different aspects of individual resource use.

The presence of small perch led to a shift in bully diet (Figure 2) accompanied by a decrease in IS (Figure 3). A population‐level diet shift in the presence of an interspecific competitor is consistent with observations across a wide range of taxa in both terrestrial and aquatic systems (e.g. Alatalo, 1981; Bonin et al., 2009; González‐Solís et al., 1997; Steinmetz et al., 2011; Young, 2004). A decrease in IS is also a common outcome of interspecific competition, though it often involves a corresponding decrease in TNW, which we did not observe. Instead, we found a change in how the population diet was partitioned, with the decrease in IS achieved by a decrease in BIC in the presence of small perch, indicating less differentiation among individuals and an increase in WIC, indicating greater individual diet breadth. This scenario does not align perfectly with any of our hypothesised outcomes. A niche shift with no decrease in TNW implies that other prey types are being relied on to replace those that have become unavailable with the competitor present. Specifically, the reduction in importance of daphniid cladocerans to bully diet in the presence of small perch may be made up for by increased reliance on existing prey such as chironomids, chydorid cladocerans and ostracods, as well as the inclusion of new prey such as mites. We speculate that reduced zooplankton availability in the presence of small perch meant that a larger component of bully population diet was made up of benthic and epibenthic prey. This might result in less among‐individual variation in foraging strategy and thus reduced BIC, even as individuals are including more prey taxa in their diets.

Competition with small perch also resulted in a population‐level shift in microhabitat use (Figure 2), although it was not accompanied by changes in IS in habitat (Figure 3). The shift was driven largely by bullies moving away from the water column and the cage in the middle of the mesocosm and toward the mesocosm walls in the presence of small perch. This shift is consistent with the diet shift which saw bullies relying less on pelagic cladocerans when small perch were present. Unlike with diet, competition did result in a near‐significant decrease in population habitat niche width and a weak but significant decrease in the average individual niche width. This pattern may simply reflect the constrained amount of space and variety of microhabitats available in the mesocosms, so that decreased reliance on some microhabitats necessarily led to some niche contraction. Niche shifts and niche contraction in response to interspecific competition are often thought to contribute to the reduction in overlap in resource use between competitors, which can promote coexistence between competing species (see for instance Koivisto et al., 2018). A broad shift away from pelagic food sources and habitats may be a general response of bullies to the presence of juvenile perch.

Contrary to predictions, predation risk did not significantly influence bully population diet (Figure 2a) or IS in diet (Figure 3A), nor did the interactive effect of competition and predation. We had hypothesised that the presence of a predator would lead either to decreased diet IS due to exclusion from riskier resources, or to increased IS if it revealed latent variation among individuals (e.g. in risk tolerance). Predation risk did not have any effects on population diet composition or population or individual diet breadth, so the treatment may have been insufficient to influence foraging patterns. No actual predation from large perch was observed during the experiment, and while it cannot be ruled out as some fish were unaccounted for, cannibalism and bird predation are also potential explanations. Bullies were observed to sometimes approach large perch in what appeared to be predator inspection behaviours, but we cannot be sure if the thrice weekly predator exposure was sufficient to influence long‐term perception of predation risk. Alternatively, as predation has the potential to both increase and decrease IS in diet (Eklöv & Svanbäck, 2006; Sharpe & Chapman, 2014), it may be less likely to have consistent effects than other drivers such as intra‐ and interspecific competition and resource diversity (Araújo et al., 2011).

Predation and competition interactively affected the degree of IS in habitat use (Figure 3B). Despite the lack of effects of predators on bully diet or on habitat composition, large perch presence did appear to influence long‐term patterns of microhabitat use. There was a decrease in IS, driven by a decrease in between‐individual variation in microhabitat (BIC), from control mesocosms to predator mesocosms (Figure 4F). The interactive effect arose because in the presence of small perch, there was no longer any effect of large perch presence. This pattern does not directly align with our hypotheses, but shares some features of the hypothesised interaction under Scenario 2 (Figure 1). Population habitat niche width (TNW) was generally lower in all treatments with perch (Figure 4B), although none of the effects reached statistical significance. The combination of decreased between‐individual variation in the presence of predators and decreased within‐individual variation in the presence of competitors likely led to the interactive effect whereby the presence of competitors negated the impact of predators on individual habitat specialisation. This is to our knowledge the first evidence that different life stages of the same antagonist can interactively affect the degree of IS in an intraguild prey species.

While competition and predation can individually or interactively alter the degree of IS in a population, the strength of that response is likely contingent on the nature of both competitors and predators. Prey responses to competitive and predatory interactions can be density‐dependent (Lichtenstein et al., 2017), linked to population structure or influenced by variation in the abundance of a third species that modifies the interaction (Chamberlain et al., 2014). These responses may also change over ontogenetic stages, implying that the effects of competition can prevail between certain size or age classes, while predation effects might be stronger between others (Persson, 1988). In the context of life history IGP, competition and predation may interact when different age classes of the same antagonists co‐occur. Within‐population dynamics leading to density shifts among age classes might therefore lead to shifts in their relative effects on the resource use of the intraguild prey population and its resulting degree of IS. Our experimental design allowed the detection of significant main and interactive effects of predation risk and competition within small intraguild prey populations. Future studies could seek to consider both density‐mediated effects of predation as well as responses to predation risk, and to assess the impacts of intraguild predators in larger and more complex ecosystems. Another potential direction for future research is to investigate whether impacts on native species' IS are a widespread but unrecognised consequence of species invasion.

Our study demonstrates that antagonistic interactions affect ecological niches at the population and the individual levels, both in isolation and in combination. In ecological communities, the impacts of IGP might be mitigated by environmental variation and by compensatory mechanisms and behavioural responses from both predator and intraguild prey. Considering individual‐level responses to both competition and predation risk can help better understand the emergent consequences of interspecific interactions within food webs and ecosystems.

## AUTHOR CONTRIBUTIONS

Marine R. A. Richarson and Travis Ingram developed the initial idea and methodology for the study. Marine R. A. Richarson collected and analysed the data. Marine R. A. Richarson wrote the manuscript. Both authors contributed critically to the drafts and gave final approval for publication.

## CONFLICT OF INTEREST STATEMENT

The authors declare no conflict of interest.

## Data Availability

Data available from the Dryad Digital Repository https://doi.org/10.5061/dryad.wdbrv161p (Richarson & Ingram, 2025).

## References

[jane70090-bib-0002] Alatalo, R. V. (1981). Interspecific competition in Tits *Parus* spp. and the Goldcrest *Regulus regulus*: Foraging shifts in multispecific flocks. Oikos, 37, 335–344.

[jane70090-bib-0003] Araújo, M. S. , Bolnick, D. I. , & Layman, C. A. (2011). The ecological causes of individual specialisation. Ecology Letters, 14, 948–958. 10.1111/j.1461-0248.2011.01662.x 21790933

[jane70090-bib-0004] Arim, M. , & Marquet, P. A. (2004). Intraguild predation: A widespread interaction related to species biology. Ecology Letters, 7, 557–564. 10.1111/j.1461-0248.2004.00613.x

[jane70090-bib-0005] Baines, C. B. , McCauley, S. J. , & Rowe, L. (2014). The interactive effects of competition and predation risk on dispersal in an insect. Biology Letters, 10, 20140287. 10.1098/rsbl.2014.0287 24919703 PMC4090550

[jane70090-bib-0006] Beleznai, O. , Tholt, G. , Tóth, Z. , Horváth, V. , Marczali, Z. , & Samu, F. (2015). Cool headed individuals are better survivors: Non‐consumptive and consumptive effects of a generalist predator on a sap feeding insect. PLoS One, 10, e0135954. 10.1371/journal.pone.0135954 26295476 PMC4546593

[jane70090-bib-0007] Bolnick, D. I. , Svanback, R. , Fordyce, J. A. , Yang, L. H. , Davis, J. M. , Hulsey, C. D. , & Forister, M. L. (2003). The ecology of individuals: Incidence and implications of individual specialization. The American Naturalist, 161, 1–28. 10.1086/343878 12650459

[jane70090-bib-0008] Bolnick, D. I. , Yang, L. H. , Fordyce, J. A. , Davis, J. M. , & Ck, R. S. (2002). Measuring individual‐level resource specialization. Ecology, 83(10), 2936–2941.

[jane70090-bib-0009] Bonin, M. C. , Srinivasan, M. , Almany, G. R. , & Jones, G. P. (2009). Interactive effects of interspecific competition and microhabitat on early post‐settlement survival in a coral reef fish. Coral Reefs, 28, 265–274. 10.1007/s00338-008-0451-y

[jane70090-bib-0010] Catry, T. , Alves, J. A. , Gill, J. A. , Gunnarsson, T. G. , & Granadeiro, J. P. (2014). Individual specialization in a shorebird population with narrow foraging niche. Acta Oecologica, 56, 56–65. 10.1016/j.actao.2014.03.001

[jane70090-bib-0011] Chamberlain, S. A. , Bronstein, J. L. , & Rudgers, J. A. (2014). How context dependent are species interactions? Ecology Letters, 17, 881–890. 10.1111/ele.12279 24735225

[jane70090-bib-0013] Costa‐Pereira, R. , Rudolf, V. H. W. , Souza, F. L. , & Araújo, M. S. (2018). Drivers of individual niche variation in coexisting species. Journal of Animal Ecology, 87, 1452–1464. 10.1111/1365-2656.12879 29938791

[jane70090-bib-0014] Cote, J. , Fogarty, S. , Tymen, B. , Sih, A. , & Brodin, T. (2013). Personality‐dependent dispersal cancelled under predation risk. Proceedings of the Royal Society B: Biological Sciences, 280, 20132349. 10.1098/rspb.2013.2349 PMC3826230 24197414

[jane70090-bib-0015] Cronin, J. T. , Haynes, K. J. , & Dillemuth, F. (2004). Spider effects on planthopper mortality, dispersal, and spatial population dynamics. Ecology, 85, 2134–2143. 10.1890/03-0591

[jane70090-bib-0016] Duncan, K. (1967). The food and population structure of perch (*Perca fluviatilis* L.) in Lake Mahinerangi. Transactions of the Royal Society of New Zealand: Zoology, 9, 45–52.

[jane70090-bib-0017] Eklöv, P. , & Svanbäck, R. (2006). Predation risk influences adaptive morphological variation in fish populations. The American Naturalist, 167, 440–452. 10.1086/499544 16673351

[jane70090-bib-0018] Fodrie, F. J. , Yeager, L. A. , Grabowski, J. H. , Layman, C. A. , Sherwood, G. D. , & Kenworthy, M. D. (2015). Measuring individuality in habitat use across complex landscapes: Approaches, constraints, and implications for assessing resource specialization. Oecologia, 178, 75–87. 25669451 10.1007/s00442-014-3212-3

[jane70090-bib-0019] Fox, J. , & Weisberg, S. (2019). An R companion to applied regression (3rd. ed.). Sage Publications.

[jane70090-bib-0020] Friendly, M. , Fox, J. , & Monette, G. (2023). heplots: Visualizing tests in multivariate linear models. R package version 1.7.5. https://CRAN.R‐project.org/package=heplots

[jane70090-bib-0021] Fulton, C. , Bellwood, D. , & Wainwright, P. (2001). The relationship between swimming ability and habitat use in wrasses (Labridae). Marine Biology, 139, 25–33. 10.1007/s002270100565

[jane70090-bib-0022] Goldsmith, R. J. (2004). Impacts of European perch Perca fluviatilis introduction on native common bully Gobiomorphus cotidianus [Unpublished doctoral dissertation]. University of Otago.

[jane70090-bib-0023] González‐Solís, J. , Oro, D. , Jover, L. , Ruiz, X. , & Pedrocchi, V. (1997). Trophic niche width and overlap of two sympatric gulls in the Southwestern Mediterranean. Oecologia, 112, 75–80. 10.1007/s004420050285 28307378

[jane70090-bib-0024] Griffiths, W. (1976). Food and feeding habits of European perch in the Selwyn River, Canterbury, New Zealand. New Zealand Journal of Marine and Freshwater Research, 10, 417–428.

[jane70090-bib-0026] Hjelm, J. , Persson, L. , & Christensen, B. (2000). Growth, morphological variation and ontogenetic niche shifts in perch (*Perca fluviatilis*) in relation to resource availability. Oecologia, 122, 190–199. 10.1007/PL00008846 28308372

[jane70090-bib-0027] Hooker, O. E. , Van Leeuwen, T. E. , & Adams, C. E. (2017). The physiological costs of prey switching reinforce foraging specialization. Journal of Animal Ecology, 86, 605–614. 10.1111/1365-2656.12632 28075009

[jane70090-bib-0029] Kerr, N. R. , & Ingram, T. (2021). Personality does not predict individual niche variation in a freshwater fish. Behavioral Ecology, 32, 159–167. 10.1093/beheco/araa117

[jane70090-bib-0030] Koivisto, E. , Hoset, K. S. , Huitu, O. , & Korpimäki, E. (2018). Habitat use of coexisting *Microtus* vole species under competition and predation risk. Canadian Journal of Zoology, 96, 237–244. 10.1139/cjz-2016-0272

[jane70090-bib-0031] Kondoh, M. (2003). Foraging adaptation and the relationship between food‐web complexity and stability. Science, 299, 1388–1391. 10.1126/science.1079154 12610303

[jane70090-bib-0032] Kristensen, E. A. , & Closs, G. P. (2004). Anti‐predator response of naïve and experienced common bully to chemical alarm cues. Journal of Fish Biology, 64(3), 643–652. 10.1111/j.1095-8649.2004.00328.x

[jane70090-bib-0033] Lichtenstein, J. L. L. , Wright, C. M. , McEwen, B. , Pinter‐Wollman, N. , & Pruitt, J. N. (2017). The multidimensional behavioural hypervolumes of two interacting species predict their space use and survival. Animal Behaviour, 132, 129–136. 10.1016/j.anbehav.2017.08.010 29681647 PMC5909842

[jane70090-bib-0034] Ludgate, B. G. , & Closs, G. (2003). Responses of fish communities to sustained removals of perch (*Perca fluviatilis*), Science for Conservation. Department of Conservation, Wellington, New Zealand.

[jane70090-bib-0035] McDowall, R. M. (1990). New Zealand freshwater fishes: A natural history and guide. Heinemann Reed MAF Publishing Group.

[jane70090-bib-0036] Oksanen, J. , Simpson, G. L. , Blanchet, F. G. , Kindt, R. , Legendre, P. , Minchin, P. R. , O'Hara, R. B. , Solymos, P. , Stevens, M. H. H. , Szoecs, E. , Wagner, H. , Barbour, M. , Bedward, M. , Bolker, B. , Borcard, D. , Carvalho, G. , Chirico, M. , Caceres, M. D. , Durand, S. , … Weedon, J. (2022). vegan: Community Ecology Package. CARN.

[jane70090-bib-0037] Persson, L. (1988). Asymmetries in competitive and predatory interactions in fish populations. In B. Ebenman & L. Persson (Eds.), Size‐structured populations (pp. 203–218). Springer Berlin Heidelberg.

[jane70090-bib-0038] Polis, G. A. , Myers, C. A. , & Holt, R. D. (1989). The ecology and evolution of intraguild predation: Potential competitors that eat each other. Annual Review of Ecology and Systematics, 20, 297–330.

[jane70090-bib-0039] Preisser, E. L. , Bolnick, D. I. , & Benard, M. F. (2005). Scared to death? The effects of intimidation and consumption in predator‐prey interactions. Ecology, 86, 501–509. 10.1890/04-0719

[jane70090-bib-0040] R Core Team . (2022). R: A language and environment for statistical computing. R Foundation for Statistical Computing.

[jane70090-bib-0041] Richarson, M. R. A. (2020). *Effects of interspecific interactions on individual specialisation* [PhD thesis, University of Otago].

[jane70090-bib-0042] Richarson, M. R. A. , & Ingram, T. (2025). Effects of competition and predation risk from a life history intraguild predator on individual specialisation. *Dryad Digital Repository*. 10.5061/dryad.wdbrv161p PMC12424273 40583217

[jane70090-bib-0043] Roughgarden, J. (1972). Evolution of niche width. The American Naturalist, 106, 683–718.

[jane70090-bib-0044] Schoener, T. W. (1974). Resource partitioning in ecological communities. Science, 185, 27–39. 17779277 10.1126/science.185.4145.27

[jane70090-bib-0045] Sharpe, D. M. T. , & Chapman, L. J. (2014). Niche expansion in a resilient endemic species following introduction of a novel top predator. Freshwater Biology, 59, 2539–2554. 10.1111/fwb.12452

[jane70090-bib-0046] Stein, A. B. , Bourquin, S. L. , & McNutt, J. W. (2015). Avoiding intraguild competition: Leopard feeding ecology and prey caching in northern Botswana. African Journal of Wildlife Research, 45, 247–257. 10.3957/056.045.0247

[jane70090-bib-0047] Steinmetz, R. , Garshelis, D. L. , Chutipong, W. , & Seuaturien, N. (2011). The shared preference niche of sympatric Asiatic black bears and sun bears in a tropical forest mosaic. PLoS One, 6, 1–11. 10.1371/journal.pone.0014509 PMC3024313 21283792

[jane70090-bib-0048] Tran, T. N. Q. , Jackson, M. C. , Sheath, D. , Verreycken, H. , & Britton, J. R. (2015). Patterns of trophic niche divergence between invasive and native fishes in wild communities are predictable from mesocosm studies. Journal of Animal Ecology, 84, 1071–1080. 10.1111/1365-2656.12360 25732893 PMC5098174

[jane70090-bib-0049] Werner, E. E. , & Peacor, S. D. (2003). A review of trait‐mediated indirect interactions in ecological communities. Ecology, 84, 1083–1100. 10.1890/0012-9658(2003)084[1083:AROTII]2.0.CO;2

[jane70090-bib-0052] Young, K. A. (2004). Asymmetric competition, habitat selection, and niche overlap in juvenile salmonids. Ecology, 85, 134–149. 10.1890/02-0402

[jane70090-bib-0053] Zaccarelli, N. , Bolnick, D. I. , & Mancinelli, G. (2013). RInSp: An R package for the analysis of individual specialization in resource use. Methods in Ecology and Evolution, 4, 1018–1023. 10.1111/2041-210X.12079

